# Evaluation of post-drowning rescue missions in the pre-hospital emergency of Mazandaran during 5 Years 

**Published:** 2022-12

**Authors:** Zoya Hadinejad, Zeinab Sajadi, Sadegh Ahmadi-Mazhin, Kobra Gholami, Leila Hosseini, Maryam Mohseni, Hassan Talebi Ghadicolaei

**Affiliations:** ^*a*^ Health in Emergency and Disaster Research Center, University of Social Welfare and Rehabilitation Sciences, Tehran, Iran.; ^*b*^ Emergency Medical Services, Mazandaran University of Medical Sciences, Sari, Iran.; ^*c*^ Department of Public Health, School of Health, Ahvaz Jundishapur University of Medical Sciences, Ahvaz, Iran.; ^*d*^ Emergency Medical Services, Mazandaran University of Medical Sciences, Sari, Iran.

## Abstract

**Background:**

Every year, many people die due to drowning and its complications as a severe health problem in Northern provinces. The first step in planning health problems in any society is to prioritize problems based on epidemiological trends. This is an epidemiological study on drowning missions performed by Mazandaran Emergency Medical Services for 5 years.

**Methods:**

This retrospective cross-sectional study was conducted on mission forms of all drowning victims in Mazandaran pre-hospital emergency from the beginning of 2017 to August 1400. The data of age, gender, drowning place, mission result (dispatch, mission cancellation, outpatient treatment, and death), and accident time and date were collected and analyzed using SPSS Software (Version 19) and the chi-square test.

**Results:**

Out of the 1127 rescued drowning cases, most incidents occurred between 10:00 AM and 12:00 PM and between 5:00 PM and 8:00 PM. A total of 720 cases (63.9%) were men and the remaining 407 cases (36.1%) were women. In addition, 506 (44.9%) cases were dispatched, 167 (14.8%) died, 341 (30.3%) were treated on-site, and the remaining 10% of missions were canceled. Most cases of drowning were in the age group of 20-30 years old. The most common place of drowning was in the unpatrolled area of the beach with 891 cases (79%). Following the COVID-19 spread and lockdown in 2020-2021, the drowning cases in the river and water channels increased so that 9 cases in 2017 reached 101 cases in August 2021.

**Conclusions:**

Most of the drowning cases occurred in unpatrolled areas among the young and productive age group of society. Development and maintenance of protected swimming beaches, as well as public education on first aid for drowning victims, seem necessary. Further, public warning and relief systems should be strengthened to make people aware of the dangers of swimming in unpatrolled and unsafe areas.

**Keywords:**

Epidemiology, Drowning, Prehospital Emergency Care

